# PhANNs, a fast and accurate tool and web server to classify phage structural proteins

**DOI:** 10.1371/journal.pcbi.1007845

**Published:** 2020-11-02

**Authors:** Vito Adrian Cantu, Peter Salamon, Victor Seguritan, Jackson Redfield, David Salamon, Robert A. Edwards, Anca M. Segall

**Affiliations:** 1 Computational Science Research Center, San Diego State University, San Diego, United States of America; 2 Viral Information Institute, San Diego State University, San Diego, United States of America; 3 Department of Mathematics and Statistics, San Diego State University, San Diego, United States of America; 4 Department of Biology, San Diego State University, San Diego, United States of America; Johns Hopkins University, UNITED STATES

## Abstract

For any given bacteriophage genome or phage-derived sequences in metagenomic data sets, we are unable to assign a function to 50–90% of genes, or more. Structural protein-encoding genes constitute a large fraction of the average phage genome and are among the most divergent and difficult-to-identify genes using homology-based methods. To understand the functions encoded by phages, their contributions to their environments, and to help gauge their utility as potential phage therapy agents, we have developed a new approach to classify phage ORFs into ten major classes of structural proteins or into an “other” category. The resulting tool is named PhANNs (Phage Artificial Neural Networks). We built a database of 538,213 manually curated phage protein sequences that we split into eleven subsets (10 for cross-validation, one for testing) using a novel clustering method that ensures there are no homologous proteins between sets yet maintains the maximum sequence diversity for training. An Artificial Neural Network ensemble trained on features extracted from those sets reached a test F_1_-score of 0.875 and test accuracy of 86.2%. PhANNs can rapidly classify proteins into one of the ten structural classes or, if not predicted to fall in one of the ten classes, as “other,” providing a new approach for functional annotation of phage proteins. PhANNs is open source and can be run from our web server or installed locally.

## Author summary

Bacteriophages (phages, viruses that infect bacteria) are the most abundant biological entity on Earth. They outnumber bacteria by a factor of ten. As phages are very different from each other and from bacteria, and we have relatively few phage genes in our database compared to bacterial genes, we are unable to assign function to 50–90% of phage genes. In this work, we developed PhANNs, a machine learning tool that can classify a phage gene as one of ten structural roles, or “other”. This approach does not require a similar gene to be known.

This is a *PLOS Computational Biology* Software paper.

## Introduction

Bacteriophages (phages) are the most abundant biological entity on the Earth [1]. They modulate microbial communities in several possible ways: by lysing specific taxonomic members or narrow groups of microbiomes, they affect the microbial population dynamics and change niche availability for different community members. Via transduction and/or lysogeny, they mediate horizontal transfer of genetic material such as virulence factors [2], metabolic auxiliary genes [3], photosystems and other genes to enhance photosynthesis[4], and phage production in general, by providing the host with immunity from killing by other phages. Temperate phages can become part of the host genome as prophages; most bacterial genomes contain at least one, and often multiple prophages [5,6].

Phage structures (virions) are composed of proteins that encapsulate and protect their genomes. The structural proteins (or virion proteins) also recognize the host, bind to its surface receptors and deliver the phage’s genome into the host’s cell. Phage proteins, especially structural ones, vary widely between phages and phage groups, so much so that sequence alignment based methods to assign gene function fail frequently: we are currently unable to assign function to 50–90% of phage genes [7]. Experimental methods such as protein sequencing, mass spectrometry, electron microscopy, or crystallography, in conjunction with antibodies against individual proteins, can be used to identify structural proteins but are expensive and time-consuming. A fast and easy-to-use computational approach to predict and classify phage structural proteins would be highly advantageous as part of pipelines for identifying functional roles of proteins of bacteriophage origins. The current increased interest in using phages as therapeutic agents [8,9] motivates annotations for as much of the phage genome as possible. Even if they are somewhat tentative and not experimentally validated, annotations of the relatively non-toxic structural proteins versus the potentially host health-threatening toxins and other virulence factors could inform decisions whether to choose one specific phage versus another.

Machine learning has been used to attack similar biological problems. In 2012, Seguritan et al. [10] developed Artificial Neural Networks (ANNs) that used normalized amino acid frequencies and the theoretical isoelectric point to classify viral proteins as structural or not structural with 85.6% accuracy. These ANNs were trained with proteins of viruses from all three domains of life. They also trained two distinct ANNs to classify phage capsid versus phage non-capsid ORFs and phage “tail associated” versus phage “non-tail-associated” ORFs. Subsequently, several groups have used different machine learning approaches to improve the accuracy of predictions. The resulting tools are summarized in **Table 1.**

**Table 1:** Summary of previous ML-based methods for classifying viral structural proteins.

Reference	Method	Target proteins	Database size	Accuracy
Seguritan et al.[10]	ANN	structural (all viruses) versus non-structural (all viruses)	6,303 structural	85.6%
			7,500 non-structural	
Seguritan et al.[10]	ANN	capsid versus non-capsid (phages only)	757 capsid	91.3%
			10,929 non-capsid	
Seguritan et al.[10]	ANN	Tail-associated versus non-tail (phages only)	2,174 tail	79.9%
			16,881 non-tail	
Feng et al.[33]	Naïve Bayes	structural versus non-structural	99 structural	79.15%
			208 non-structural	
Zhang et al.[34]	Ensemble Random Forest	structural versus non-structural	253 structural	85.0%
			248 non-structural	
Galiez et al.[11]	SVM	capsid versus non-capsid	3,888 capsid	96.8%
			4,071 non-capsid	
Galiez et al.[11]	SVM	tail versus non-tail	2,574 tail	89.4%
			4,095 non-tail	
Manavalan et al.[35]	SVM	structural versus non-structural	129 structural	87.0%
			272 non-structural	
This work	ANN	Ten distinct phage structural classes plus “others”	168,660 structural	86.2%
			369,553 non-structural	

Each of these previous approaches has important limitations: 1) The classification is limited to two classes of proteins (e.g.,”capsid” or “not capsid”). 2) Training and testing sets were small (only a few hundred proteins in some cases), limiting the utility of these approaches beyond those proteins used in testing. 3) Methods that rely on predicting secondary structure (e.g., VIRALpro [11]) are slow to run. In general, these newer methods have improved accuracy at the cost of lengthening the time required for training, or have used very small training and/or test sets.

Artificial Neural Networks (ANN) are proven universal approximators of functions in ℝ^n^ [12], including the mathematical function that maps features extracted from a phage protein sequence to its structural class. We have constructed a manually-curated database of phage structural proteins and have used it to train a feed-forward ANN to assign any phage protein to one of eleven classes (ten structural classes plus a catch-all class labeled "others"). Furthermore, we developed a web server where protein sequences can be uploaded for classification. The full database, as well as the code for PhANNs and the webserver, are available for download at http://edwards.sdsu.edu/phanns and https://github.com/Adrian-Cantu/PhANNs

## Methods

### Database

In this work, we generated two complementary protein databases, "classes" and "others". The "classes" database contains curated sequences of ten phage structural functions (Major capsid, Minor capsid, Baseplate, Major tail, Minor tail, Portal, Tail fiber, Tail sheath, Collar, and Head-Tail Joining). These functional classes are not exhaustive (and we will add more classes in the future); they represent the dominant structural protein roles present in most (but not all) phages [13]. The terms/descriptors for these classes are addressed in the next section. Major capsid proteins are those that form the phage head. Many but not all phages also encode minor capsid proteins that decorate and/or stabilize the head or proteins present at the vertices of the icosahedral heador at the center of the hexon faces. Portals form a ring at the base of the phage head and serve to dock the packaging complex that translocates the genome into the phage head. Head-tail joining (aka head-tail connector or head completion) proteins form rings inserted between the portal ring and the tail. The collar is present in some phages, *e*.*g*. the Lactococcal phages, at the base of the neck/top of the tail to which the so-called whiskers attach. Major tail proteins form the inner tail tube of the tailed phages, whereas the tail sheath (aka the tail shaft) proteins form the outside of the tail, and permit contraction. Minor tail proteins may comprise several kinds of proteins associated with the tail, including the tape measure protein. Baseplate proteins are those that are attached to the tail and to which the tail fibers are attached, the latter being a relatively common determinant of host range. The "others" database contains all phage ORFs that do not encode proteins annotated as “structural” or as any of the ten categories above.

### The database of "classes"

Sequences from the ten structural classes were downloaded from NCBI's protein database using a custom search for the class title (the queries are in the “ncbi_get_structural.py” script in the GitHub repository). Curation consisted of grouping sequences by their description (part of the fasta header) and deciding which descriptions to include. The list of included headers for each class can be found here https://github.com/Adrian-Cantu/PhANNs/tree/master/model_training/01_fasta; the variations of terms included are too many to be included here. All the terms preceded by a “+” (or “+ +”) were included in the respective database. In the particular case of tail fibers, we did not include the descriptions “phage tail fiber assembly protein” (3,662 proteins) nor many “partial protein” variations (1,500+ proteins).

This method for collecting data has the limitation that a proportion of phage sequences in the database are misannotated and that NCBI has no controlled vocabulary for bacteriophage protein functions so it is occasionally difficult to account for misspelled annotations and/or alternative naming. However, it is clear from previous machine learning applications that a larger number of training examples is more important for optimal model performance than a perfectly curated training set [14]. To minimize inclusion of wrongly annotated protein sequences, we manually curated the databases to address these limitations.

### The "others" database

To generate a database for the "others" class, all available phage genomes (8,238) were downloaded from GenBank on 4/13/19. ORFs were found using the GenBank PATRIC [15] server with the phage recipe [16]. Sequences annotated as structural or any of the ten classes were removed during manual curation. Furthermore, the remaining sequences were de-replicated at 60% together with sequences in the “classes” database using CD-hit [17]. Any phage ORF that clustered with a sequence from the "classes" database was removed from the "others" database.

### Training, test, and validation split

**Fig 1:**
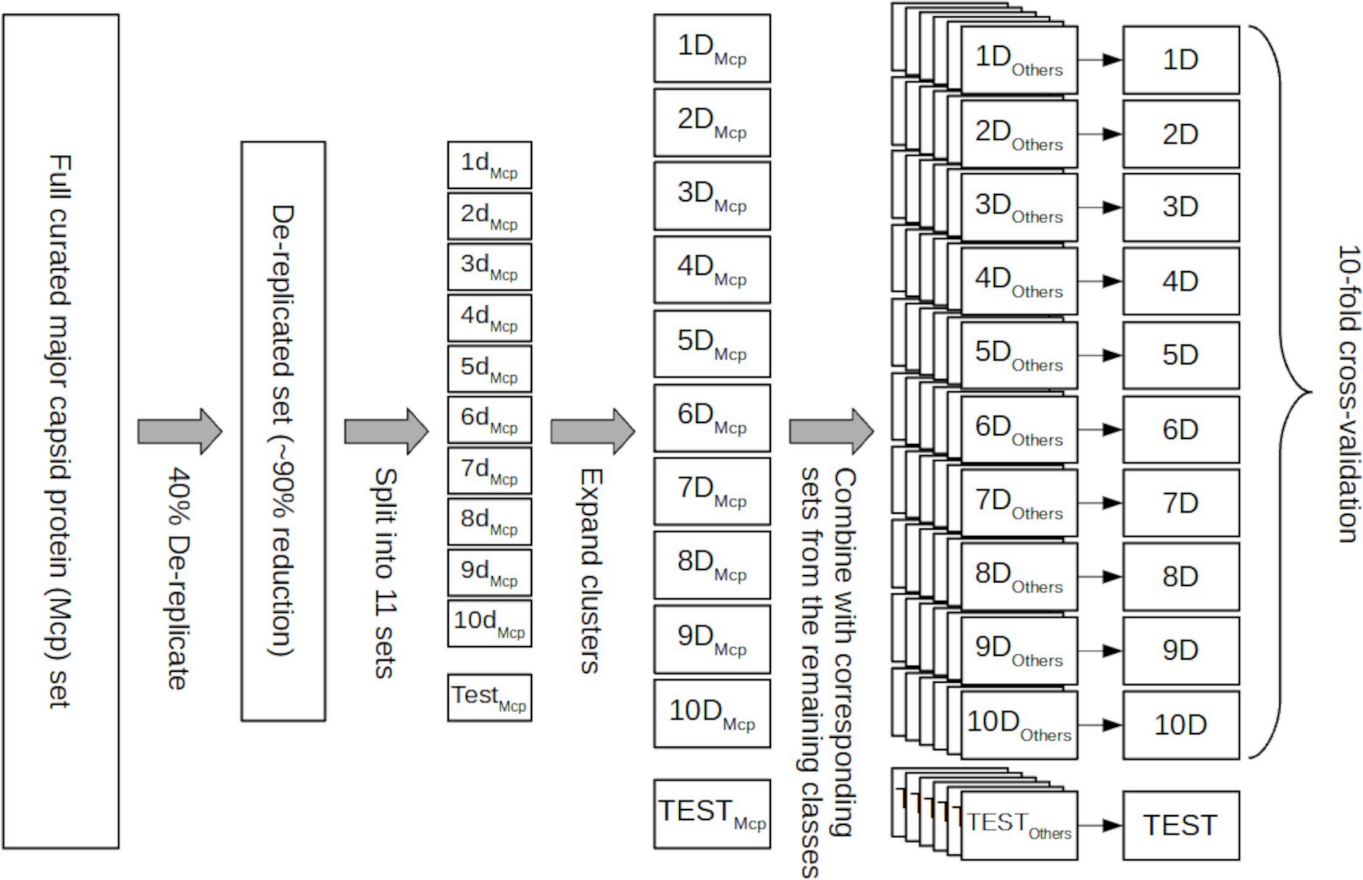
Non homologous database split—To ensure that no homologous sequences are shared between the test, validation, and training sets the sequences from each class (Major capsid proteins in this figure) were de-replicated at 40%. In the de-replicated set, no two proteins have more than 40% identity and each sequence is a representative of a larger cluster of related proteins. The de-replicated set is then randomly partitioned into eleven equal size subsets, (1d_Mcp_-10d_Mpc_ plus Test_Mpc_). Those subsets are expanded by replacing each sequence with all the sequences in the cluster it represents (subsets 1D_Mpc_-10D_Mpc_ plus TEST_Mpc_). Analogous subsets are generated for the remaining ten classes and corresponding subsets are combined to generate the subsets used for 10-fold cross-validation and testing (1D-10D and TEST).

Sequences in each class were clustered at 40% using CD-hit and split into eleven sets (10 for cross validation and one for testing, as shown in **Fig 1**). Once the clusters were established, to prevent loss of the sequence diversity available within the clusters, which is essential for optimal training, the clusters were expanded by adding back *within* each set all the representatives of that set (described in **Fig 1**). Subsequently, the sets corresponding to each structural class were merged. We named the generated sets 1D-10D and TEST. Splitting the database this way ensures that the different sets share no homologous proteins while recapturing all the sequence diversity present in each class. Finally, 100% dereplication was performed to remove identical sequences (See **Table 2**). The effect of the cluster expansion on performance is explored in **S1 and S2 Figs**.

**Table 2:** Database numbers—Raw sequences were downloaded using a custom script available at https://github.com/Adrian-Cantu/PhANNs. All datasets can be downloaded from the web server. *Numbers before and after removing sequences at least 60% identical to a protein in the classes database.

Class	Raw sequences	After manual curation	After de-replication at 40%	After expansion and de-replication at 100%
Major capsid	112,987	105,653	1,945	35,755
Minor capsid	2,901	1,903	261	1,055
Baseplate	75,599	19,293	401	6,221
Major tail	66,513	35,030	536	7,704
Minor tail	94,628	80,467	918	18,002
Portal	210,064	189,143	2,310	59,745
Tail fiber	29,132	18,514	1,222	7,256
Tail sheath	37,885	35,570	599	15,349
Collar	4,224	3,709	339	2,105
Head-Tail joining	60,270	58,658	1,317	15,468
**Total structural**	**694,203**	**547,940**	**9,848**	**168,660**
Others	733,006	643,735/643,380*	106,004	369,553

### Extraction of features

The frequency of each dipeptide (400 features) and tripeptide (8,000 features) was computed for each ORF sequence in both the “classes” and “others” databases. As a potential time-saving procedure during neural net training while also permitting classification of more diverse sequences, each amino acid was assigned to one of seven distinct "side chain" chemical groups (**S1 Table**). The frequency of the "side chain" 2-mers (49 features), 3-mers (343 features), and 4-mers (2,401 features) was also computed. Finally, some extra features, namely isoelectric point, instability index (whether a protein is likely to degrade rapidly; [18]), ORF length, aromaticity (relative frequency of aromatic amino acids; [19]), molar extinction coefficient (how much light the protein absorbs) using two methods (assuming reduced cysteins or disulfide bonds), hydrophobicity, GRAVY index (average hydropathy; [20]) and molecular weight, were computed using Biopython [21]. All 11,201 features were extracted from each of 538,213 protein sequences. The complete training data set can be downloaded from the web server (https://edwards.sdsu.edu/phanns).

### ANN architecture and training

We used Keras [22] with the TensorFlow [23] back-end to train eleven distinct ANN models using a different subset of features. We named the models to indicate which feature sets were used in training: the composition of 2-mers/dipeptides (di), 3-mers /tripeptides (tri) or 4-mer/tetrapeptide (tetra), or side chain groups (sc) (as shown in **S1 Table**), and whether we included the extra features (p) or not. A twelfth ANN model was trained using all the features (**Table 3**).

**Table 3:** Feature types included in each of the 12 models. **di**—2-mer/dipeptide composition; **tri**—3-mer/tripeptide composition; **tetra**—4-mer/tetrapeptide composition; **sc**—side-chain grouping; **p**—plus all the extra features [isoelectric point, instability index (whether a protein is likely to be degraded rapidly), ORF length, aromaticity (relative frequency of aromatic amino acids), molar extinction coefficient (how much light a protein absorbs) using two methods (assuming reduced cysteines or disulfide bonds), hydrophobicity, GRAVY index (average hydropathy), and molecular weight, as computed using Biopython. **-** *Per class score figures are available as supplementary material.

Model	di	tri	di_sc	tri_sc	tetra_sc	p
di_sc*			x			
di_sc_p*			x			x
tri_sc*				x		
tri_sc_p*				x		x
tetra_sc*					x	
tetra_sc_p*					x	x
di	x					
di_p	x					x
tri		x				
tri_p		x				x
tetra_sc_tri_p		x			x	x
all	x	x	x	x	x	x

Each ANN consists of an input layer, two hidden layers of 200 neurons, and an output layer with 11 neurons (one per class). A dropout function with 0.2 probability was inserted between layers to prevent overfitting. ReLU activation (to introduce non-linearity) was used for all layers except the output, where softmax was used. Loss was computed by categorical cross-entropy and the ANN is trained using the "opt" optimizer until 10 epochs see no training loss reduction. The model at the epoch with the lowest validation loss is used. Class weights inversely proportional to the number of sequences in that class were used.

#### 10-fold cross-validation

**Fig 2:**
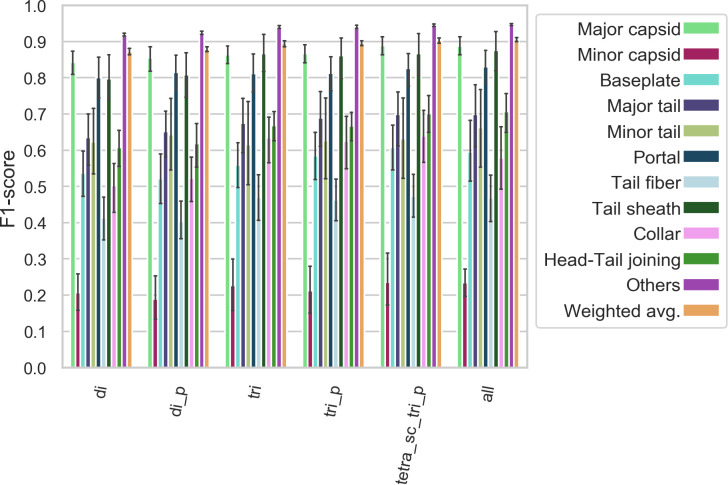
Model-specific F_1_ score—F_1_ scores (harmonic mean of precision and recall) for each polypeptide model/class combination. All models follow similar trends as to which classes are more or less difficult to classify correctly. Error bars represent the 95% confidence intervals.

**Fig 3:**
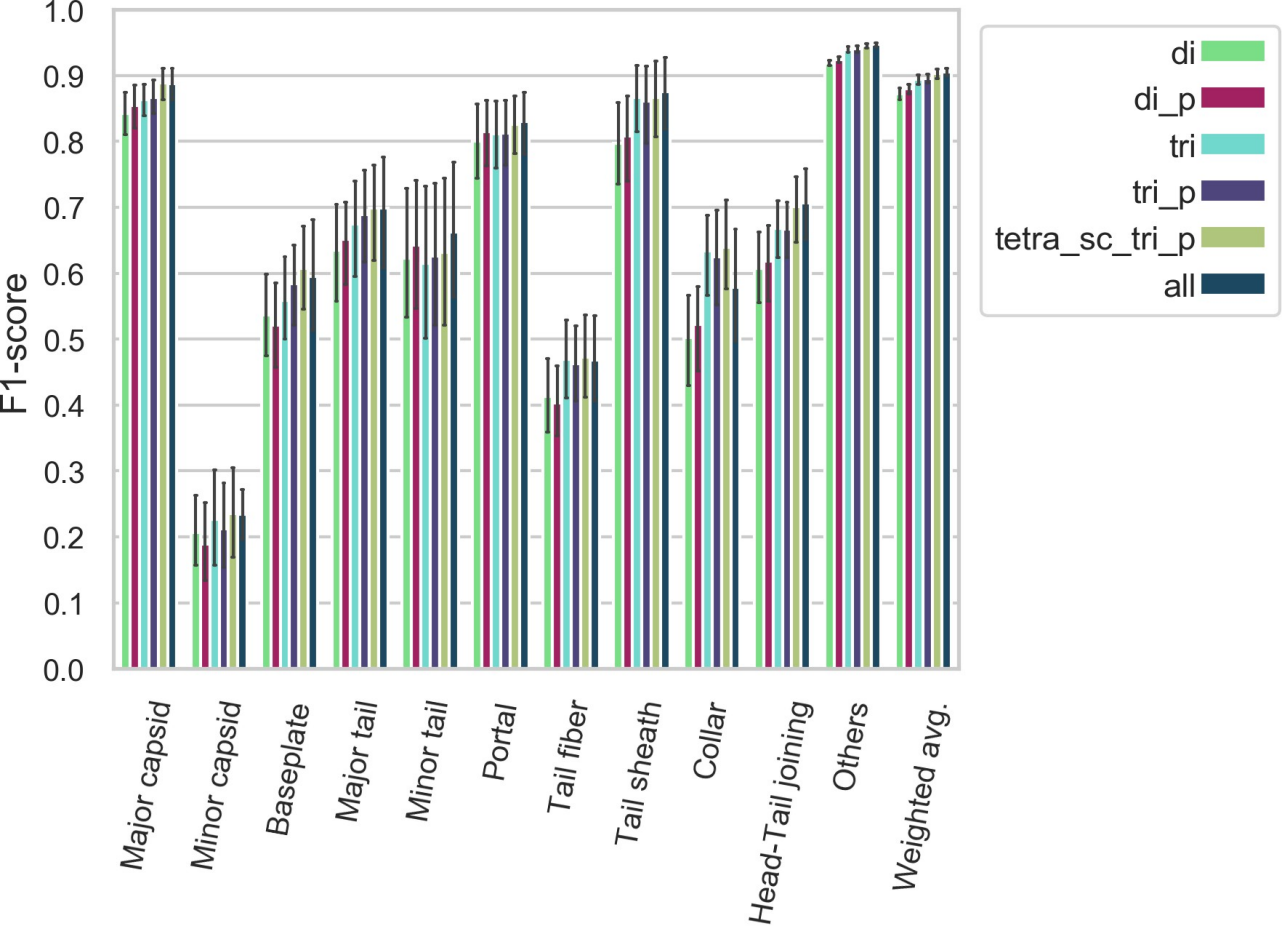
Class-specific F_1_ score—F_1_ scores (harmonic mean of precision and recall) for each polypeptide model/class combination. Some classes, such as minor capsid, tail fiber, or minor tail, are harder to classify correctly irrespective of the model used. Error bars represent the 95% confidence intervals.

**Fig 4:**
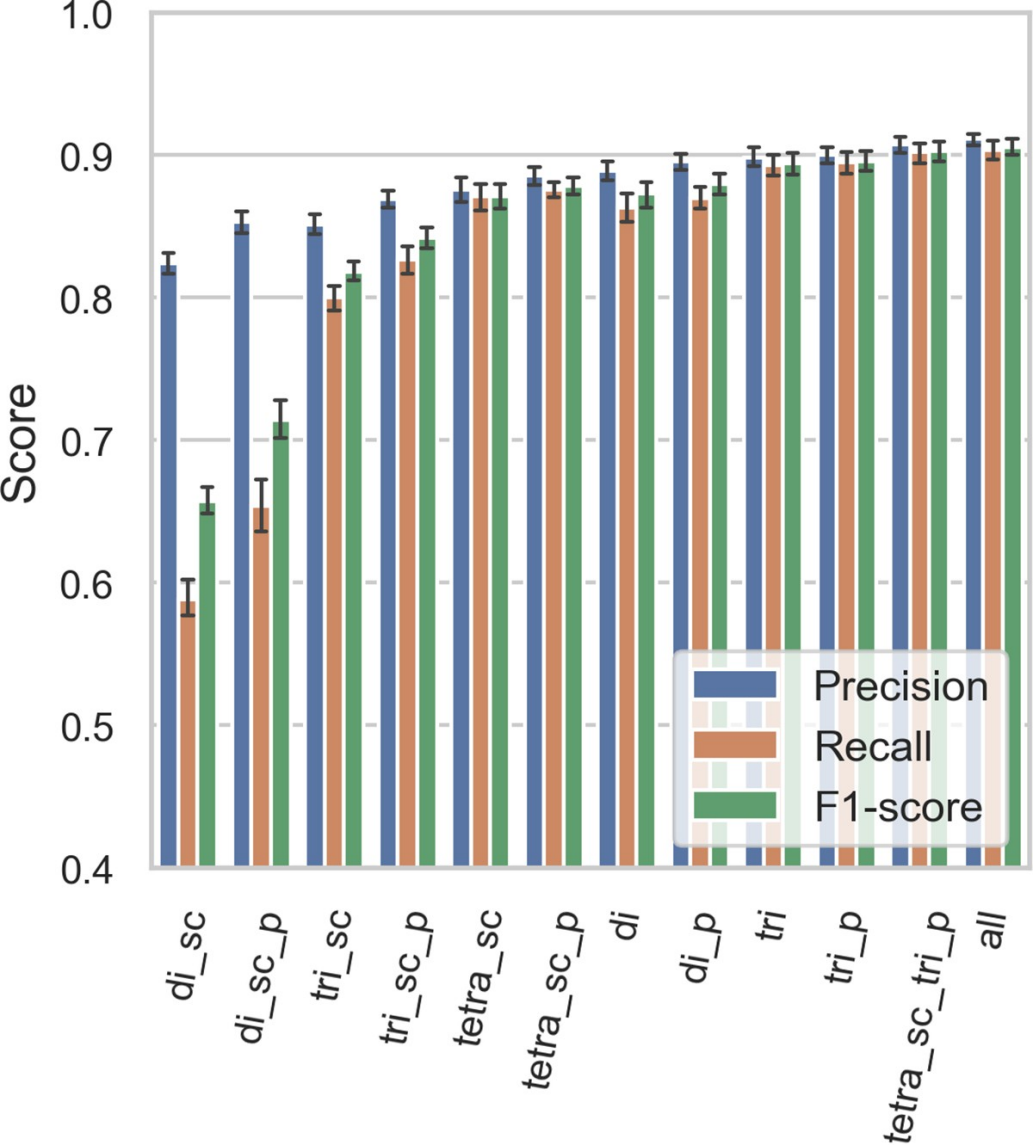
Model-specific validation weighted average scores—Precision, recall, and F_1_ scores for all models. Precision is higher in all models as the “others” class is the largest and easiest to classify correctly. Error bars represent the 95% confidence intervals.

Sets 1D to 10D (see **Fig 1**) were used to perform 10-fold cross-validation; ten ANNs were trained as described above, sequentially using one set as the validation set and the remaining nine as the training set. The results are summarized in **Figs 2, 3, 4, S1 and S2.**

#### The PhANNs score

For each input sequence, PhANNs run 10 ANNs predictions (those trained during the 10-fold cross-validation). Each of those 10 ANNs outputs the soft-max scores for every class (a number between 0 and 1, such that the score of all classes adds to 1). PhANNs outputs the per class sum of the ten ANNs scores (the maximum achievable PhANNs score is 10, as there are ten ANNs). The input sequence is classified as the class with the highest PhANNs score.

**Fig 5:**
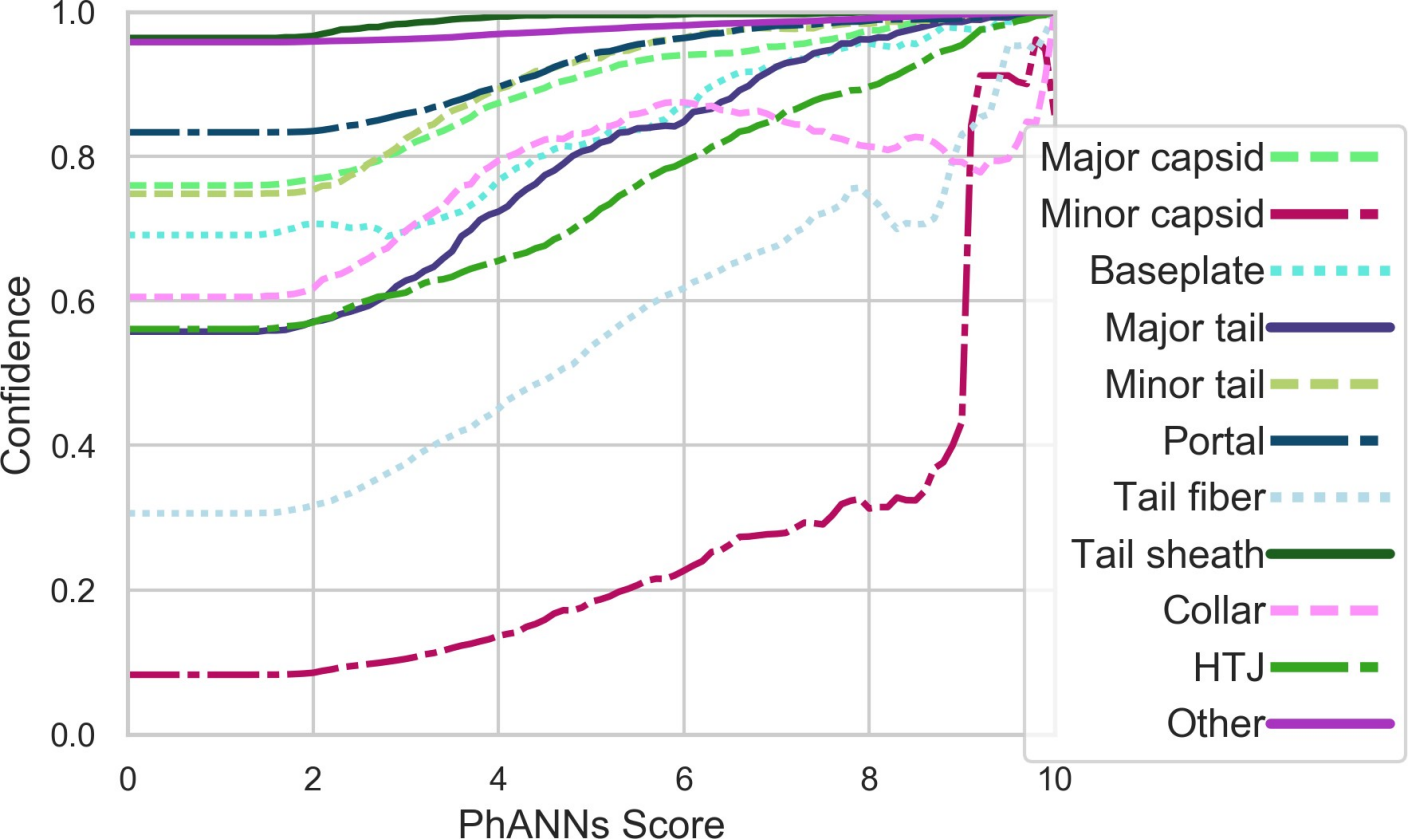
Per class relationship between PhANNs score and confidence—The confidence corresponding to a particular class PhANNs score represents the fraction of true positives (correctly classified) sequences in the test set that were classified as that class, with a given PhANNs score or higher. As it is uncommon for the highest class PhANNs score to be less than 2, the left side of the graph includes all test proteins that were classified as that class, and the confidence corresponds to the per class precision (see Table 4).

To give a clearer indication of the quality of this prediction we added a “confidence” score to each prediction. The “confidence” score shows what fraction of sequences in the test set that were classified as the same class as the input sequence, and with the same PhANNs score or higher, were correctly classified (True positives). The confidence scores differ depending on the protein class. For example, a sequence classified as “major capsid” with a PhANNs score of 7 has 97% confidence, while a “Tail fiber” with a PhANNs score of 7 has only 82.4% confidence. The per class relationship between the PhANNs score and the confidence is explored in **Fig 5**.

### Web server

We developed an easy-to-use web server for users to upload and classify their own sequences. Although ANNs need substantial computational resources for training the model (between 54,861 and 127,756,413 parameters need to be tuned, depending on the model), the trained model can make fast *de novo* predictions. Our web server (https://edwards.sdsu.edu/phanns) can predict the structural class of an arbitrary protein sequence in seconds and assign all the ORFs in a phage genome to one of the 10 classes in minutes. The application can also be downloaded and run locally for large numbers of queries or if privacy is a concern.

## Results and discussion

We evaluated the performance of 120 ANNs (10 per model type) on their respective validation set. For each ANN, we computed the precision, recall, and F_1_-score of the 11 classes. A “weighted average” precision, recall and F_1_-score, where the score for each class is weighted by the number of proteins in that class (larger classes contribute more to the score) was computed. The accuracy (fraction of observation correctly classified) is equivalent to the weighted average recall. The three weighted average scores are represented as a 12th class. This gives us ten observations for each combination of model type and class, which allows us to construct the confidence intervals depicted in **Figs 2, 3 and 4.**

**Fig 6:**
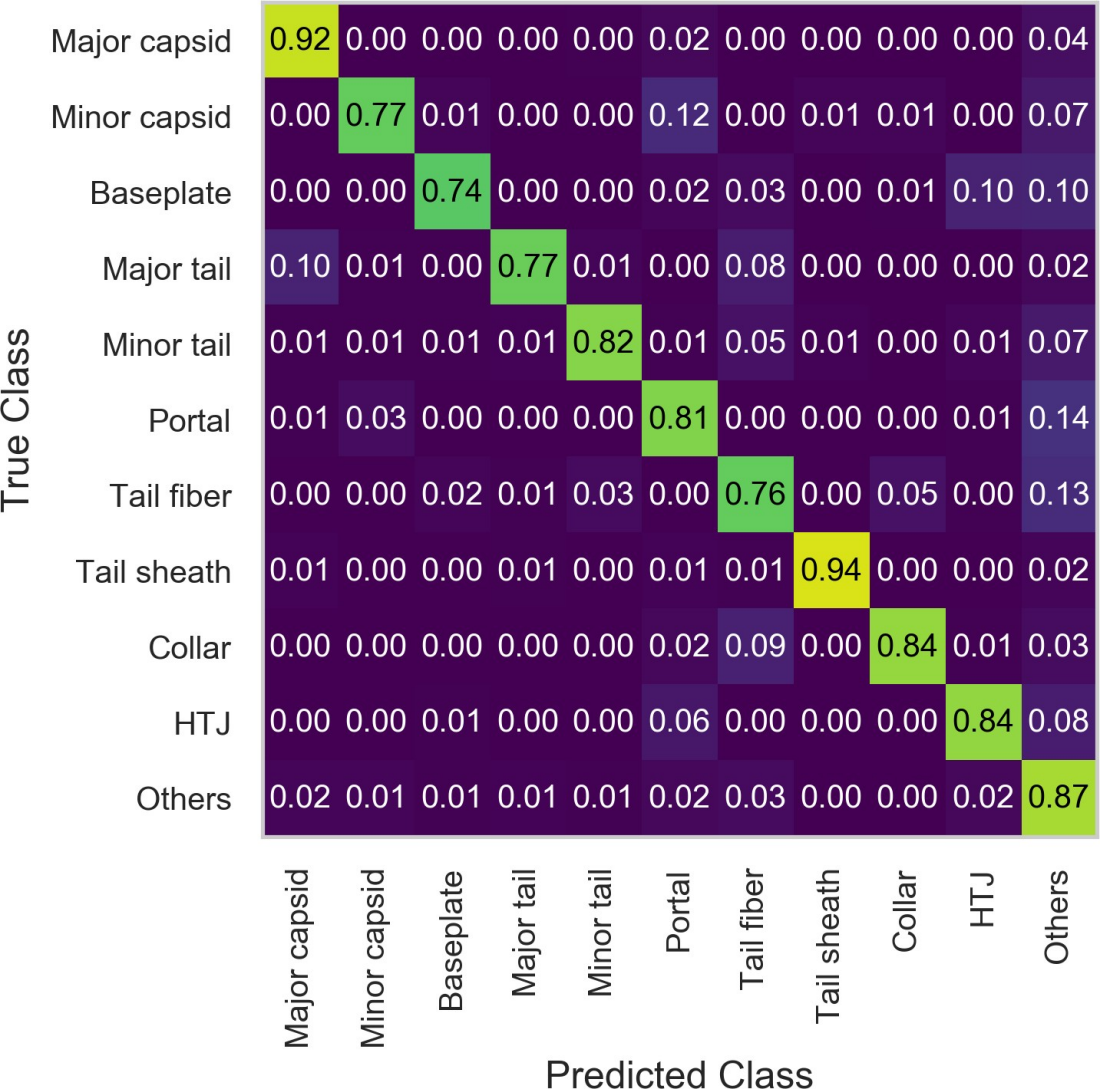
Confusion matrix using the “tetra_sc_tri_p” model—Each row shows the proportional classification of test sequences from a particular class. A perfect classifier would have 1 on the diagonal and 0 elsewhere. In general, a protein that is misclassified is predicted as “others”.

**(Figs 2 and S1)** shows that all the models follow the same trend as to which classes they predict with higher or lower accuracy. Some classes of proteins, for example major capsids, collars, and head-tail joining proteins, are predicted with high accuracy. On the other hand, the minor capsid and tail fiber protein classes seem to be intrinsically hard to predict with high accuracy irrespective of the model type used (**Figs 3 and S2**). One reason for this is the limited size of the training set: the minor capsid protein set is the smallest class, with only 581 proteins available for inclusion in our database. Even if the classes were weighted according to their size during training, it appears we do not have enough training examples to identify this class with high accuracy. Furthermore, “minor capsid” is often misclassified as “portal” (**Fig 6**). This probably reflects an annotation bias, as we found about 800 proteins annotated as “portal (minor capsid)” in the raw sequences. When the ~800 proteins are analyzed with PhANNs, over 90% are predicted to be portal proteins. Although these were removed during manual curation of the training data sets, some (small) fraction of minor capsid proteins in our database may have been annotated as “minor capsid” by homology to one of those 800 sequences.

The predictive accuracy for a specific class of proteins is likely to be affected by the bias in the training datasets. The bias could be biological and/or due to a sampling bias. An example of the former is the tail fiber class: the tail fiber is one of the determinants of the host range of the virus, and is under strong evolutionary selective pressure [24–29]. On the other hand, sampling bias may be introduced due to oversampling of certain types of phages, such as the thousands of mycobacterial phages isolated as part of the SEA-PHAGES project [30], many of which are highly related to each other.

Average validation F_1_-scores range from 0.653 for the “di_sc” model to 0.841 for the “tetra_sc_tri” model (**Fig 4**). Although the average validation F_1_-score for the top three models “tri_p” (0.832), “tetra_sc_tri_p” (0.841), and “all” (0.827) are not significantly different from each other, we decided to use “tetra_sc_tri_p” for the web server and all subsequent analyses because, while it uses ~7% fewer features than “all” (10,409 vs 11,201), we expect that the tetra side chain features may be better than the tripeptide features at generalizing predictions and accessing greater sequence diversity.

Using the “tetra_sc_tri_p” ensemble, we predicted the class of each sequence in the test set (46,801) by averaging the scores of each of the ten ANNs. Results are summarized in **Fig 6 and Table 4.** Doing this we reach a test F_1_-score of 0.89 and accuracy of 86.2% over the eleven classes.

**Table 4:** Results of per class classification for the test set. Support indicates the number of test sequences in each specific class. accuracy (fraction of observation correctly classified) is equivalent to the weighted average recall (weighted by the support of each class). The macro average is unweighted (all classes contribute the same).

	precision	recall	f1-score	support
**Major capsid**	0.80	0.91	0.85	2,456
**Minor capsid**	0.07	0.78	0.13	81
**Baseplate**	0.69	0.75	0.72	851
**Major tail**	0.55	0.79	0.65	502
**Minor tail**	0.66	0.82	0.73	1,072
**Portal**	0.81	0.81	0.81	5,261
**Tail fiber**	0.35	0.74	0.47	648
**Tail sheath**	0.97	0.93	0.95	2,031
**Collar**	0.51	0.86	0.64	300
**Head-Tail joining**	0.56	0.84	0.67	1,277
**Others**	0.96	0.86	0.91	32,322
**macro avg**	0.63	0.83	0.68	46,801
**weighted avg**	0.89	0.86 (accuracy)	0.87	46,801

**Fig 7:**
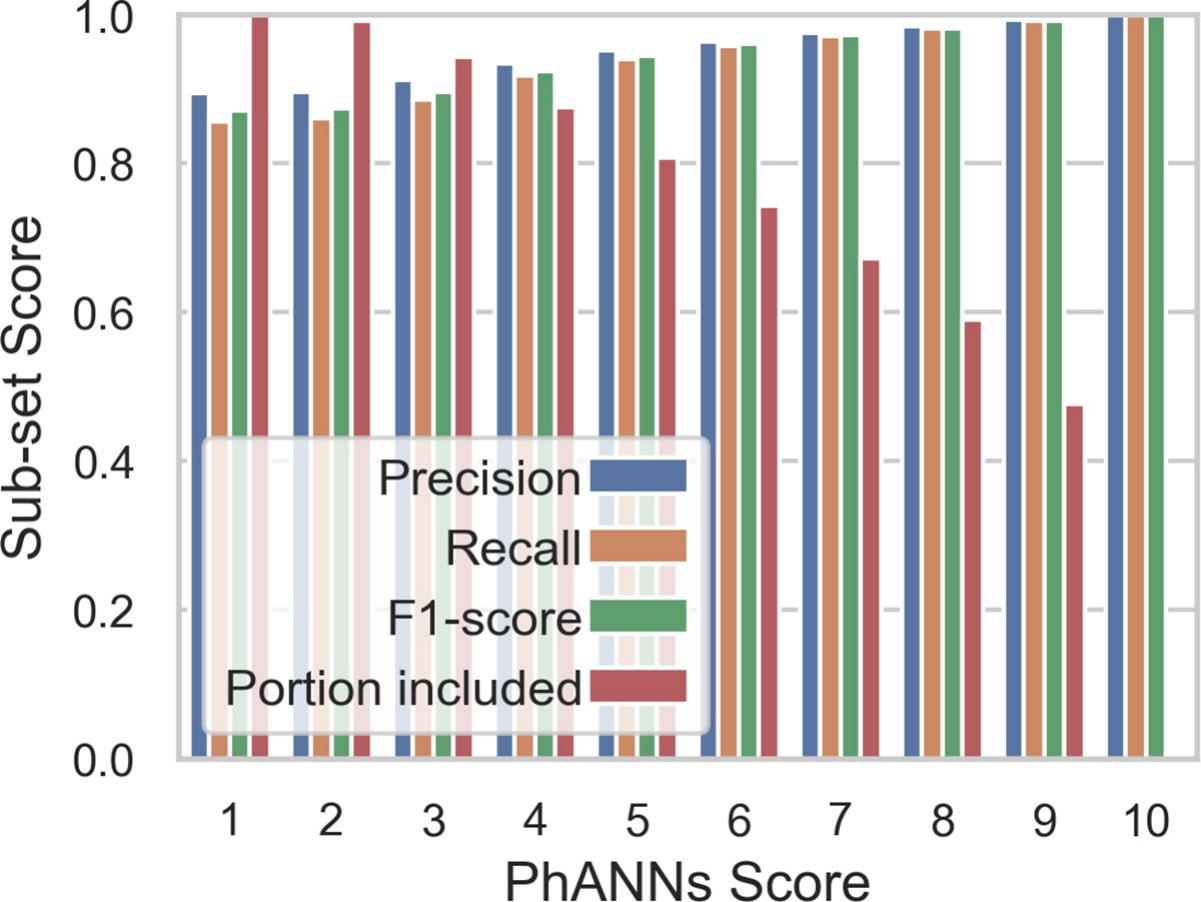
Effect of disregarding low scoring test proteins—Progression of the weighted average precision, recall and F_1_-score of the test set after excluding low scoring proteins. The portion of included proteins is the fraction that can be classified if you only trust that score or higher. Very few test proteins have PhANNs score of 10 and not all classes are represented.

Higher accuracy can be reached if one is willing to disregard sequences with low PhANNs scores. Using only sequences with a PhANNs score of 5 or higher, the F_1_-score for the test set is 0.945, accuracy is 94%, with 9,006 of 46,801 (~20%) test sequences being “not classified”. If using sequences with a PhANNs score of 8 or higher, the F_1_-score for the test set is 0.982, accuracy is 98%, but 19,208 of 46,801 (~41%) test sequences would be “not classified” (see **Fig 7**). **Table 4** shows summary statistics for the complete test set, while **Table 5** shows the same statistics for the test subset of sequences with PhANNs 8 or greater. The stringency with which users interpret the PhANNs score may vary depending on their specific need. Therefore we recommend that the actual PhANNs score (or the confidence score) be reported in addition to the predicted function class.

**Table 5:** Results of per class classification for proteins in the test set with a PhANNs score of 8 or higher. Support indicates the number of test sequences in each specific class. accuracy (fraction of observation correctly classified) is equivalent to the weighted average recall (weighted by the support of each class). The macro average is unweighted (all classes contribute the same).

	precision	recall	F_1_-score	support
**Major capsid**	0.99	0.99	0.99	1,563
**Minor capsid**	0.28	0.96	0.43	45
**Baseplate**	0.97	0.83	0.89	151
**Major tail**	0.95	0.97	0.96	307
**Minor tail**	0.95	0.99	0.97	625
**Portal**	0.99	0.94	0.97	3,810
**Tail fiber**	0.89	0.94	0.91	360
**Tail sheath**	1.00	1.00	1.00	1,495
**Collar**	0.82	1.00	0.90	98
**Head-Tail joining**	0.91	1.00	0.95	916
**Others**	0.99	0.99	0.99	18,223
**macro avg**	0.89	0.96	0.91	27,593
**weighted avg**	0.98	0.98 (accuracy)	0.98	27,593

**Fig 8:**
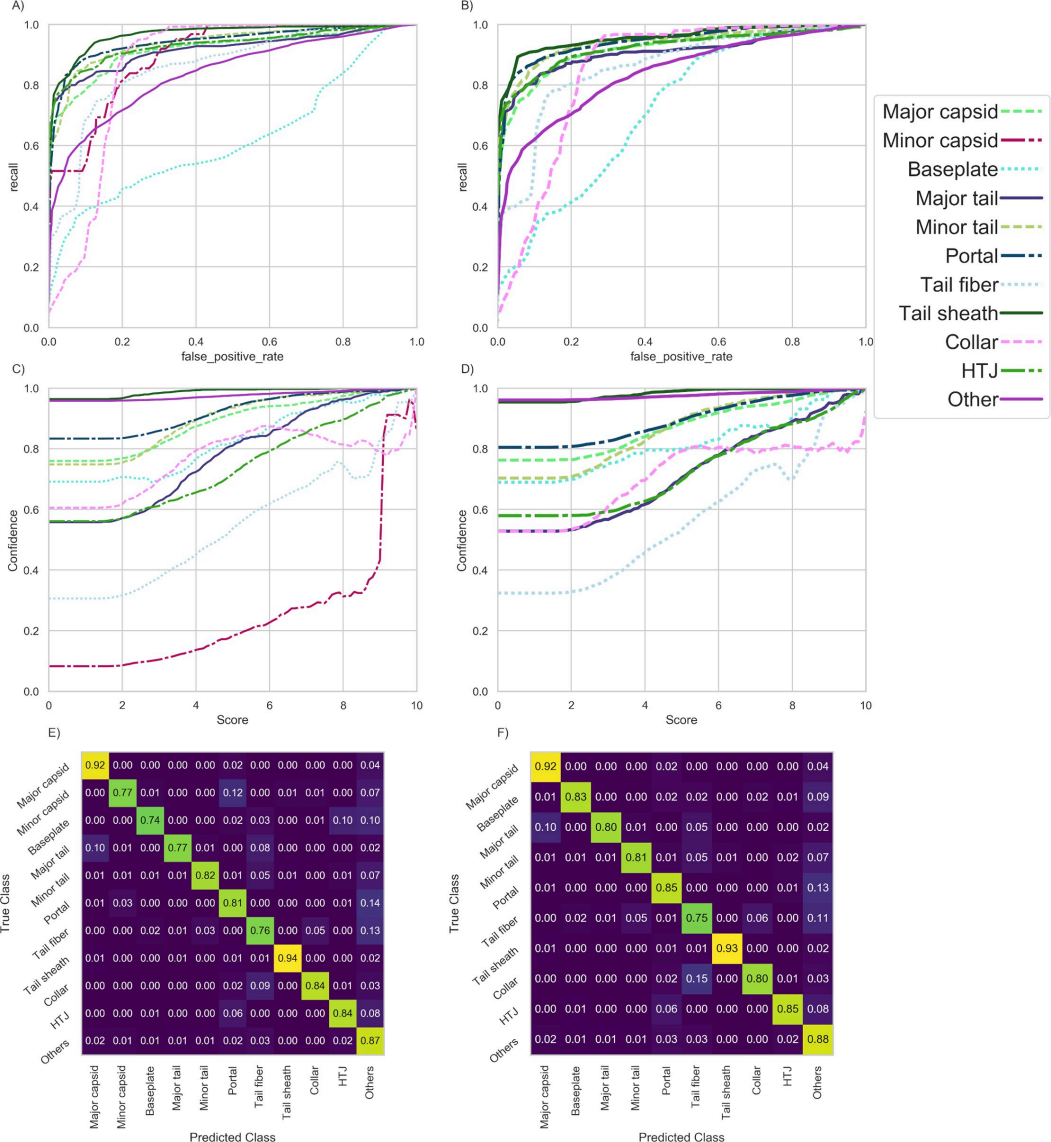
Comparison of “tetra_sc_tri_p” model trained with and without the Minor capsid class—As minor capsid is the worst performing class in our test set, we trained an analogous ANN ensemble with it removed. Panels A and B show the ROC curves for the models with and without minor capsid respectively. Panels C and D show the relationship between PhANNs score and Confidence for the models with and without minor capsid respectively. Panels E and F show the confusion matrix for the models with and without minor capsid respectively.

Because “minor capsid” is the worst performing class in our test set, we trained an analogous ANN ensemble without that class to explore if accuracy of the remaining classes is improved. Multiple metrics can be used to assess which model is better. The per class ROC curves of both models [**Fig 8A (with minor capsid class) and 8-B (without minor capsid class**)] and areas under the curves are similar. Removing the minor capsid class from the models doesn't significantly alter the relationship between the PhANNs score and the confidence score (**Fig 8C and 8D**). The confusion matrices of both models (**Fig 8E and 8F**) show that predictions for portal proteins improve, as 3% of them are misclassified as minor capsid. For all other classes, the two models are similar with respect to which classes are most commonly confused. A comparison of per class precision, recall and F_1_-score can be found in **Table 6.** When the minor capsid class is excluded, metrics are just as likely to improve as to worsen, and the accuracy gain is only 1%; greater accuracy gains can be achieved by disregarding sequences with low PhANNs scores as “not classified,” as described above. Therefore, we decided not to exclude the minor capsid class from our model; the performance in this class is likely to improve in the future, as more sequences become available and, hopefully, are experimentally validated.

**Table 6:** The effect on the models’s scores from excluding the minor capsid class (mc)—Most scores are affected only slightly and are as likely to improve as to worsen.

	precision	precision (mc)	recall	recall (mc)	F_1_-score	F_1_-score (mc)	support	ROC area	ROC area (mc)
**Major capsid**	0.76	0.76	0.92	0.92	0.83	0.83	2456	0.917	0.918
**Minor capsid**	0.08	-	0.77	-	0.15	-	81 (0)	0.899	-
**Baseplate**	0.69	0.69	0.74	0.83	0.72	0.75	851	0.621	0.72
**Major tail**	0.56	0.53	0.77	0.80	0.65	0.64	502	0.918	0.91
**Minor tail**	0.75	0.70	0.82	0.81	0.78	0.75	1070	0.939	0.94
**Portal**	0.83	0.80	0.81	0.85	0.82	0.82	5261	0.943	0.945
**Tail fiber**	0.31	0.32	0.76	0.75	0.44	0.45	648	0.861	0.86
**Tail sheath**	0.96	0.95	0.94	0.93	0.95	0.94	2031	0.986	0.957
**Collar**	0.61	0.53	0.84	0.80	0.70	0.63	300	0.865	0.85
**HTJ**	0.56	0.58	0.84	0.85	0.67	0.69	1277	0.933	0.923
**Others**	0.96	0.96	0.87	0.88	0.91	0.92	33402	0.838	0.838
**macro avg**	0.64	0.68	0.83	0.84	0.69	0.74	47879 (47798)		
**weighted avg**	0.90	0.90	0.86	0.87	0.88	0.88	47879 (47798)		

We compared the performance of PhANNs with that of VIRALpro by predicting the function class of each other's test set. Doing this requires us to map our 11 classes onto VIRALpro’s 4 (capsid versus not-capsid, tail versus not tail). We decided not to use the PhANNs “collar” or “baseplate” test set as VIRALpro has a hard time classifying them (presumably because it was not trained on those classes). Hence we discarded any of the VIRALpro test proteins that PhANNs predicted as “collar” or “baseplate”. “Capsid” in VIRALpro means either “major capsid” or “minor capsid” in PhANNs. “Tail” in VIRALpro means “Major tail”, “Minor tail”, “Tail fiber” or “Tail sheath” in PhANNs. This transformation makes possible the comparison of the two tools. Results are summarized in **Table 7.** The two tools have similar accuracy, with VIRALpro slightly better at predicting capsid proteins and PhANNs slightly better at predicting tail proteins. It is important to mention that the VIRALpro predictions took several days on a 200+ CPU cluster (it would take a few years on a laptop). A similarly sized test takes less than an hour using the PhANNs server.

**Table 7:** Comparison of PhANNs with VIRALpro. Results from using VIRALpro test set in PhANNs and PhANNs test set in VIRALpro.

	PhANNs test set in TAILpro	TAILpro test set in PhANNs	PhANNs test set in CAPSIDpro	CAPSIDpro test set in PhANNs
test set size	10,805	672	15,107	787
precision	0.28	0.77	0.14	0.82
recall	0.79	0.68	0.86	0.32
accuracy	0.80	0.82	0.70	0.67
F1-score	0.42	0.72	0.25	0.46

The utility of the PhANNs tool is to permit more extensive function predictions of metagenome sequences from phages used for phage therapy (A. Cobian, N. Jacobson, M. Rojas, H. Hamza, R. Rowe, D. Conrad, and A. Segall, et al., work in progress) and to better describe the coding potential of the virome in patients suffering from diseases such as inflammatory bowel disease versus household controls (A. Segall, R. Edwards, A. Cantu, S. Handley, and D. Wang, work in progress). In some cases, phage-associated sequences from isolated viromes have no or very weak functional predictions when using BLAST, RPS-BLAST, or related bioinformatic tools (work in progress). In parallel, we are experimentally validating some of the predicted functions using electron microscopy and X-ray crystallography (S.H. Hung, V. Seguritan, et al., ms. in preparation).

The performance of any machine learning system is limited by the availability and cost of training examples [14]. Invariably, top performing image and audio classification systems must augment their training data with synthetic examples created by applying semantically orthogonal transformations to the training set (i.e., slightly rotating or distorting an image, or adding background noise to audio) [31,32]. In bioinformatics, the current practice of de-replication moves us in exactly the opposite direction—perfectly good samples cannot be used if their overlap with other samples is too high, leaving only one version of the biostring to use for training, thereby ignoring sequence variations. This despite the fact that biological examples such as protein sequence data are replete with variations from a consensus sequence or motif. Our approach overcomes this failing by using *all* non-redundant data. By splitting the dataset into the training, validation, and test sets after first de-replicating at 40%, we remove even slightly redundant samples and make sure that none of the performance is due to data memorization rather than generalization. Augmenting the training set by expanding the clusters to include all non-redundant samples is the novel idea we have introduced in the present paper as a way of increasing our training set size and hence our accuracy.

## Conclusion

ANNs are a powerful tool to classify phage structural proteins when homology-based alignments do not provide useful functional predictions, such as “hypothetical” or “unknown function”. This approach will become more accurate as more and better characterized phage structural protein sequences, especially more divergent ones, are experimentally validated and become available for inclusion in our training sets. This method can also be applied to predicting the function of unknown proteins of prophage origin in bacterial genomes. In the future, we plan to expand this approach to more protein classes and to viruses of eukaryotes and archaea.

## Supporting information

S1 Table Side chain groupings. (XLS)

S1 Fig Model-specific F_1_ score—F_1_ scores (harmonic mean of precision and recall) for each side chain model/class combination. All models follow similar trends as to which classes are more or less difficult to classify correctly. Error bars represent the 95% confidence intervals.
(PNG)

S2 Fig Class-specific F_1_ score—F_1_ scores (harmonic mean of precision and recall) for each side chain model/class combination. Some classes, such as minor capsid, tail fiber, or minor tail, are harder to classify correctly irrespective of the model used. Error bars represent the 95% confidence intervals.
(PNG)

S3 Fig Comparison of the validation weighted average F_1_-score of three models on the same feature sets—We compared our ANN ensemble trained on 1D-10D sets against a logistic regression trained on the 1D-10D sets and an ANN ensemble trained on the 1d-10d sets (40% dereplication, without cluster expansion—see Methods). The ANN ensembles perform significantly better than the logistic regression. Error bars represent 0.95 confidence intervals.
(PNG)

S4 Fig Per class comparison of the validation F_1_-score of three models on the “tetras_sc_tri_p feature” set—In the structural classes, the 1D-10D ANN ensemble performs slightly better than the logistic regression and significantly better than the 1d-10d ANN ensemble. In the “others” class (by far the largest), 1D-10D ANN ensemble performs as well as 1d-10d ANN and better than logistic regression. Error bars represent 0.95 confidence intervals.
(PNG)

## References

[1] Cobián Güemes AG, Youle M, Cantú VA, Felts B, Nulton J, Rohwer F. Viruses as Winners in the Game of Life. Annu Rev Virol. 2016 9 29;3(1):197–214. 10.1146/annurev-virology-100114-054952 27741409

[2] Waldor MK, Mekalanos JJ. Lysogenic conversion by a filamentous phage encoding *cholera* toxin. Science. 1996 6 28;272(5270):1910–4. 10.1126/science.272.5270.1910 8658163

[3] Breitbart M, Bonnain C, Malki K, Sawaya NA. Phage puppet masters of the marine microbial realm. Nat Microbiol. 2018 7;3(7):754–66. 10.1038/s41564-018-0166-y 29867096

[4] Frank JA, Lorimer D, Youle M, Witte P, Craig T, Abendroth J, et al Structure and function of a cyanophage-encoded peptide deformylase. ISME J. 2013 6;7(6):1150–60. 10.1038/ismej.2013.4 23407310 PMC3660681

[5] Knowles B, Silveira CB, Bailey BA, Barott K, Cantu VA, Cobián-Güemes AG, et al Lytic to temperate switching of viral communities. Nature. 2016 3 24;531(7595):466–70. 10.1038/nature17193 26982729

[6] Kang HS, McNair K, Cuevas DA, Bailey BA, Segall AM, Edwards RA. Prophage genomics reveals patterns in phage genome organization and replication. bioRxiv. 2017 3 7;114819.

[7] Edwards RA, Rohwer F. Viral metagenomics. Nat Rev Microbiol. 2005 6;3(6):504–10. 10.1038/nrmicro1163 15886693

[8] McCallin S, Sacher JC, Zheng J, Chan BK. Current State of Compassionate Phage Therapy. Viruses. 2019 4;11(4):343 10.3390/v11040343 31013833 PMC6521059

[9] Hesse S, Adhya S. Phage Therapy in the Twenty-First Century: Facing the Decline of the Antibiotic Era; Is It Finally Time for the Age of the Phage? Annu Rev Microbiol. 2019;73(1):155–74. 10.1146/annurev-micro-090817-062535 31185183

[10] Seguritan V, Alves N Jr., Arnoult M, Raymond A, Lorimer D, Burgin AB Jr., et al Artificial Neural Networks Trained to Detect Viral and Phage Structural Proteins. PLoS Comput Biol. 2012;8(8). 10.1371/journal.pcbi.1002657 22927809 PMC3426561

[11] Galiez C, Magnan CN, Coste F, Baldi P. VIRALpro: A tool to identify viral capsid and tail sequences. Bioinformatics. 2016;32(9):1405–7. 10.1093/bioinformatics/btv727 26733451 PMC5860506

[12] Csáji BC. Approximation with Artificial Neural Networks. 2001;45.

[13] Veesler D, Cambillau C. A common evolutionary origin for tailed bacteriophage functional modules and bacterial machineries. Micr Mol Biol Rev. 2011;75(3):423–33. 10.1128/MMBR.00014-11 21885679 PMC3165541

[14] Halevy A, Norvig P, Pereira F. The Unreasonable Effectiveness of Data. IEEE Intell Syst. 2009 3;24(2):8–12.

[15] Wattam AR, Davis JJ, Assaf R, Boisvert S, Brettin T, Bun C, et al Improvements to PATRIC, the all-bacterial Bioinformatics Database and Analysis Resource Center. Nucleic Acids Res. 2017 04;45(D1):D535–42. 10.1093/nar/gkw1017 27899627 PMC5210524

[16] McNair K, Zhou C, Dinsdale EA, Souza B, Edwards RA. PHANOTATE: a novel approach to gene identification in phage genomes. Bioinforma Oxf Engl. 2019 4 25; 10.1093/bioinformatics/btz265 PMC6853651 31329826

[17] Li W, Godzik A. Cd-hit: a fast program for clustering and comparing large sets of protein or nucleotide sequences. Bioinforma Oxf Engl. 2006 7 1;22(13):1658–9. 10.1093/bioinformatics/btl158 16731699

[18] Guruprasad K, Reddy BVB, Pandit MW. Correlation between stability of a protein and its dipeptide composition: a novel approach for predicting in vivo stability of a protein from its primary sequence. Protein Eng Des Sel. 1990 12 1;4(2):155–61. 10.1093/protein/4.2.155 2075190

[19] Lobry JR, Gautier C. Hydrophobicity, expressivity and aromaticity are the major trends of amino-acid usage in 999 Escherichia coli chromosome-encoded genes. Nucleic Acids Res. 1994 8 11;22(15):3174–80. 10.1093/nar/22.15.3174 8065933 PMC310293

[20] Kyte J, Doolittle RF. A simple method for displaying the hydropathic character of a protein. J Mol Biol. 1982 5 5;157(1):105–32. 10.1016/0022-2836(82)90515-0 7108955

[21] Cock PJA, Antao T, Chang JT, Chapman BA, Cox CJ, Dalke A, et al Biopython: freely available Python tools for computational molecular biology and bioinformatics. Bioinforma Oxf Engl. 2009 6 1;25(11):1422–3. 10.1093/bioinformatics/btp163 19304878 PMC2682512

[22] Chollet F, others. Keras [Internet]. 2015. Available from: https://keras.io

[23] Abadi Martín, Agarwal Ashish, Barham Paul, Brevdo Eugene, Chen Zhifeng, Citro Craig, et al TensorFlow: Large-Scale Machine Learning on Heterogeneous Systems [Internet]. 2015 Available from: https://www.tensorflow.org/

[24] Drexler K, Dannull J, Hindennach I, Mutschler B, Henning U. Single mutations in a gene for a tail fiber component of an Escherichia coli phage can cause an extension from a protein to a carbohydrate as a receptor. J Mol Biol. 1991 6 20;219(4):655–63. 10.1016/0022-2836(91)90662-p 1829115

[25] Desplats C, Krisch HM. The diversity and evolution of the T4-type bacteriophages. Res Microbiol. 2003 5;154(4):259–67. 12798230 10.1016/S0923-2508(03)00069-X

[26] Medhekar B, Miller JF. Diversity-generating retroelements. Curr Opin Microbiol. 2007 8;10(4):388–95. 10.1016/j.mib.2007.06.004 17703991 PMC2703298

[27] Ciezki K, Murfin K, Goodrich-Blair H, Stock SP, Forst S. R-type bacteriocins in related strains of *Xenorhabdus bovienii*: Xenorhabdicin tail fiber modularity and contribution to competitiveness. FEMS Microbiol Lett. 2017;364(1). 10.1093/femsle/fnw235 27737947

[28] Akusobi C, Chan BK, Williams ESCP, Wertz JE, Turner PE. Parallel Evolution of Host-Attachment Proteins in Phage PP01 Populations Adapting to Escherichia coli O157:H7. Pharm Basel Switz. 2018 6 20;11(2). 10.3390/ph11020060 29925767 PMC6027323

[29] Benler S, Cobián-Güemes AG, McNair K, Hung S-H, Levi K, Edwards R, et al A diversity-generating retroelement encoded by a globally ubiquitous *Bacteroides* phage. Microbiome. 2018 23;6(1):191 10.1186/s40168-018-0573-6 30352623 PMC6199706

[30] Jordan TC, Burnett SH, Carson S, Caruso SM, Clase K, DeJong RJ, et al A Broadly Implementable Research Course in Phage Discovery and Genomics for First-Year Undergraduate Students. mBio [Internet]. 2014 2 4 [cited 2019 Nov 13];5(1). Available from: https://www.ncbi.nlm.nih.gov/pmc/articles/PMC3950523/ 10.1128/mBio.01051-13 24496795 PMC3950523

[31] Kanda N, Takeda R, Obuchi Y. Elastic spectral distortion for low resource speech recognition with deep neural networks. In: 2013 IEEE Workshop on Automatic Speech Recognition and Understanding. 2013. p. 309–14.

[32] Ciregan D, Meier U, Schmidhuber J. Multi-column deep neural networks for image classification. In: 2012 IEEE Conference on Computer Vision and Pattern Recognition. 2012. p. 3642–9.

[33] Feng P-M, Ding H, Chen W, Lin H. Naïve bayes classifier with feature selection to identify phage virion proteins. Comput Math Methods Med. 2013;2013 10.1155/2013/530696 23762187 PMC3671239

[34] Zhang L, Zhang C, Gao R, Yang R. An ensemble method to distinguish bacteriophage virion from non-virion proteins based on protein sequence characteristics. Int J Mol Sci. 2015;16(9):21734–58. 10.3390/ijms160921734 26370987 PMC4613277

[35] Manavalan B, Shin TH, Lee G. PVP-SVM: Sequence-based prediction of phage virion proteins using a support vector machine. Front Microbiol. 2018;9(MAR). 10.3389/fmicb.2018.00476 29616000 PMC5864850

