# Supplementary figures and images for: PhANNs, a fast and accurate tool and web server to classify phage structural proteins
Source: PLoS Comput Biol. 2020 Nov 2;16(11):e1007845. doi: 10.1371/journal.pcbi.1007845 (PMC7660903; doi:10.1371/journal.pcbi.1007845)

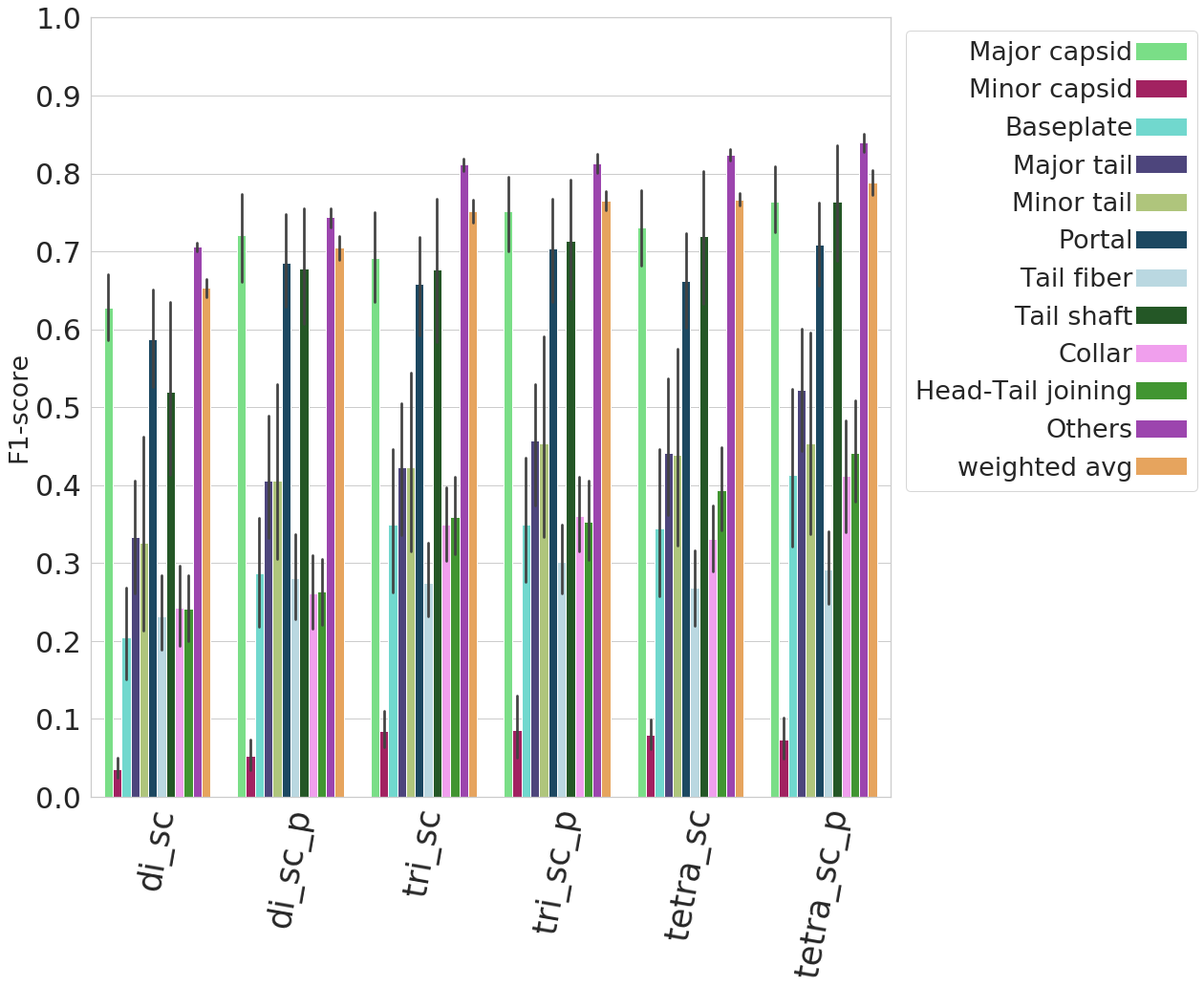

Supplement: S1 Fig — All models follow similar trends as to which classes are more or less difficult to classify correctly. Error bars represent the 95% confidence intervals. (PNG) [file pcbi.1007845.s002.png]

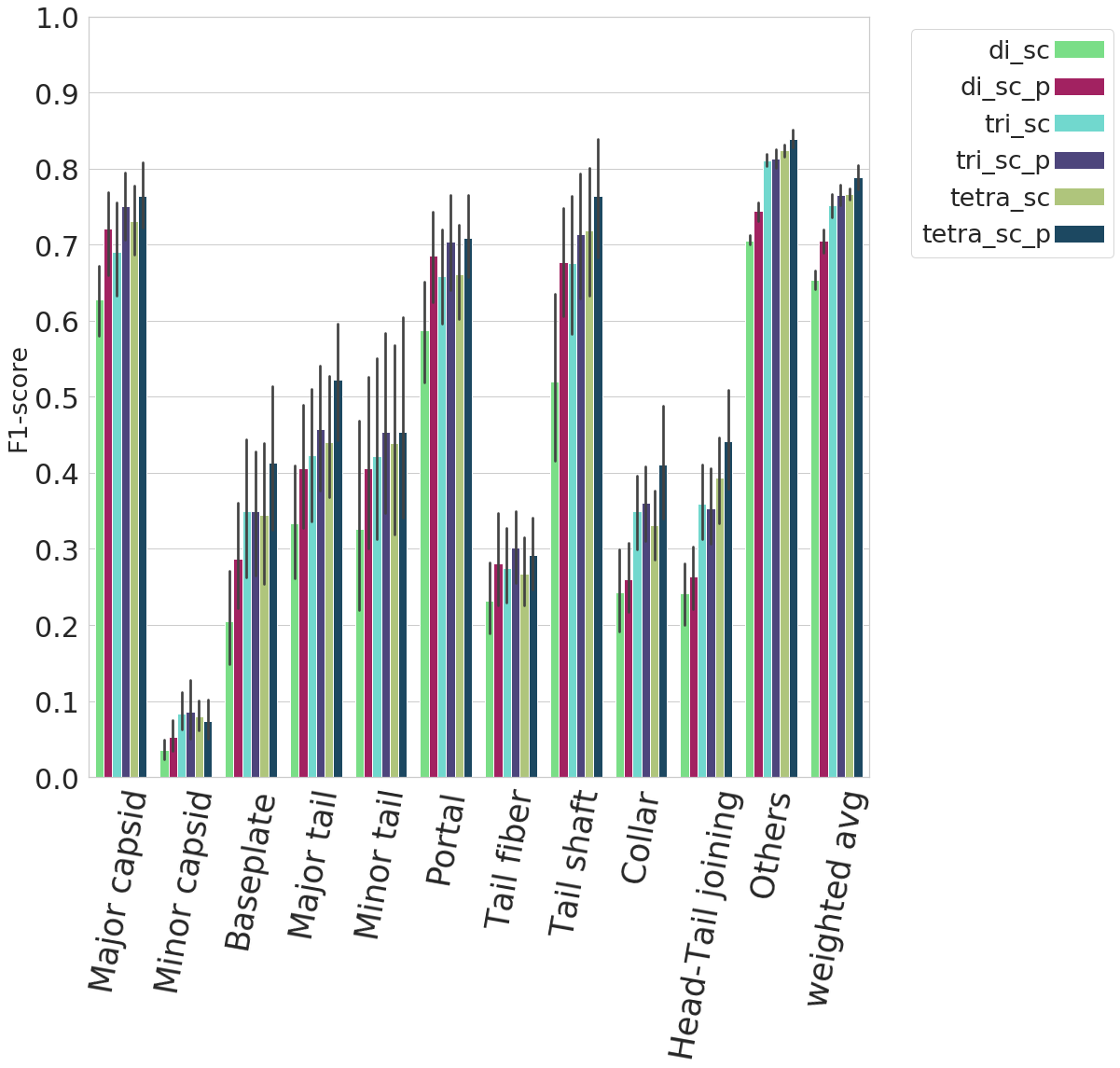

Supplement: S2 Fig — Some classes, such as minor capsid, tail fiber, or minor tail, are harder to classify correctly irrespective of the model used. Error bars represent the 95% confidence intervals. (PNG) [file pcbi.1007845.s003.png]

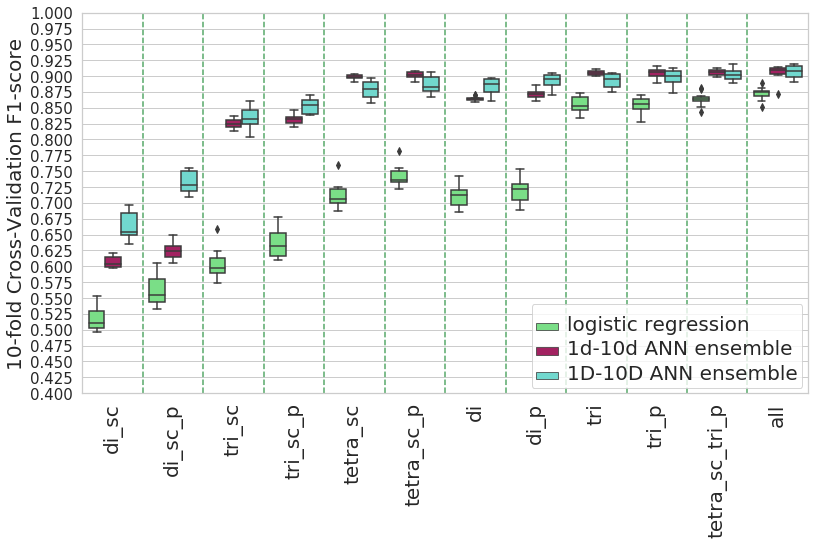

Supplement: S3 Fig — The ANN ensembles perform significantly better than the logistic regression. Error bars represent 0.95 confidence intervals. (PNG) [file pcbi.1007845.s004.png]

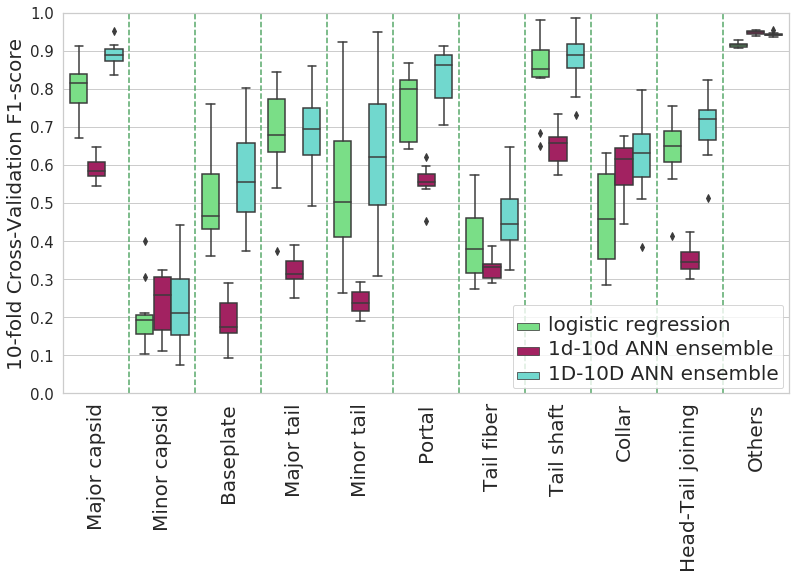

Supplement: S4 Fig — In the “others” class (by far the largest), 1D-10D ANN ensemble performs as well as 1d-10d ANN and better than logistic regression. Error bars represent 0.95 confidence intervals. (PNG) [file pcbi.1007845.s005.png]
