# Supplementary material for: PhANNs, a fast and accurate tool and web server to classify phage structural proteins
Source: PLoS Comput Biol. 2020 Nov 2;16(11):e1007845. doi: 10.1371/journal.pcbi.1007845 (PMC7660903; doi:10.1371/journal.pcbi.1007845)
Supplement: Attachment — Submitted filename: PhANNs_reviewers_responses.docx [file pcbi.1007845.s006.docx]

Dear Dr. Amaya Reik,

We very much appreciate the thoroughness of the reviewers and their helpful comments on our manuscript. We hope we have addressed all of their concerns, as summarized below. We also greatly appreciate your editorial handling of our manuscript, and look forward to your final decision.

Sincerely,

Adrian Cantu and Anca Segall

***Comments to the Authors:***

***Please note here if the review is uploaded as an attachment.***

*Reviewer #1: Cantu et al. present the tool PhANNs for the prediction of phage genes. This tool is greatly needed as the number of phage genomes being detected in metagenomic studies continues to increase and the majority of the genes are unable to be annotated. Having now tested the tool, my comments re: the manuscript can be divided into (1) the tool/code repo and (2) the manuscript. Overall, the manuscript is well written and clear.*

*Tool:*

***1. Question: Both the Webserver and GitHub repo rely on this manually-curated database. As is a concern frequently with new tools, how do the authors plan to keep this database up to date?***

We plan on updating the database (both including newly released sequences and updating manual curation) it yearly. We have funding to do this for at least four years. The database is curated by deciding which sequence descriptions to include and the set of descriptions (curating list) used in the paper is included in the github repository. Thus anyone can download an updated set of raw sequences and use the same curation lists to update the database for their own use. Details on how to do that can be found here https://github.com/Adrian-Cantu/PhANNs/tree/master/model_training

***2. Question: It looks like there is a way for the user/community to update the model file and/or create their own. For building the database and model, I'm assuming it's in the model_training folder, but there is no ReadMe describing the files in there.***

We added documentation for all parts of the model training folder. In particular, we added instructions on how to update the database, perform the curation, or both, as well as some suggestions on how to add new classes.

*https://github.com/Adrian-Cantu/PhANNs/tree/master/model_training)*

***3. The web service results list a table. It's great to be able to download it, but there is no details on how to interpret this table. What numbers are being reported by the web? Also, why are some cells highlighted on the web results? Does it automatically pick the highest score or is there some way that it distinguishes a good prediction? This information should be provided on the webpage, either with the results or the submission page. The web service is an attractive tool for less computational users. It however lacks documentation.***

We added a new section to the manuscript (lines 189-203) that describes the interpretation of the PhANNs score and how to derive a classification and confidence from it. We also added two figures: Figure 6 shows the relationship between the PhANNs score and the confidence for each class, and Figure 7 shows the effect on accuracy of excluding low scoring predictions.

For each input sequence, PhANNs run 10 ANNs predictions (those trained during the 10-fold cross-validation). Each of those 10 ANNs outputs the soft-max scores for every class (a number between 0 and 1, such that the score of each class adds to 1). PhANNs outputs the per class sum of the ten ANNs scores (the PhANNs score). The input sequence is classified as the class with the highest PhANNs score, and is highlighted in yellow.

To give a clearer indication of the quality of this prediction we added a “confidence” score to each prediction. The “confidence” score shows what fraction of test set sequences with a specific PhANNs score or higher were correctly classified (true positives). For example, a sequence classified as “major capsid” with a PhANNs score of 7 has 97% confidence that the classification is correct, while a “Tail fiber” with a PhANNs score of 7 has only 82.4% confidence that the classification is correct.

***4. As a test to see how well the tool worked with non-phage sequences, I fed it some bacterial protein sequences and was surprised to find predictions other than "Other". Some had scores of 10.00, of course without knowing what the number being reported is and what the range is, is that good? I'm imagining a scenario where a user has a contig they think is viral but are uncertain.***

First, bacterial genomes that contain either complete or partial prophages are expected to give positive scores in the case of those genes that encode structural components, as also reported in our previous paper^1^. In fact, PhANNs training data includes prophage proteins from bacterial genomes. Second, as reported in the new Table 4, the recall of the “Other” class is about 86%. If you ask PhANNs to classify non-phage proteins, about 14% will be classified as structural phage proteins, mostly tail fibers and major capsid (see Figure 5). The confidence score that is now associated with each prediction makes it easier to judge if a classification is trustworthy. If users trust only predictions with a PhANNs score of 8 or better, the “Other” recall increases to 99% — only 1% of those predictions would be expected to be misclassified. Third, it is possible that phage structural proteins may share structural features with bacterial proteins possessing other functions, for example tail proteins may share similarity with proteins that assemble into pili or transporter proteins; the PhANNs scores will reflect this structural similarity.

*Manuscript:*

***1. Details re: the prediction models are not thoroughly discussed. As this is the computational contribution of this work, additional details and testing would strengthen the paper. Related, I would consider moving Table S2 into the main text. There is no other mention of the codes used for the model names and what the individual models consider in the main text. This makes interpretation of Figure 2 challenging. Alternatively, the Figure 2 legend should include details re: what each of the model names listed on the x axis mean.***

We moved the old Table S1 to the main text (now Table 3) and added discussion on the model score (lines 189-203)), the effect of excluding minor capsid from the model (269-284), and a table describing all the scores of the tetra_sc_tri_p model.(Table 4)

***2. While the authors acknowledge that there are other tools for phage function prediction, the manuscript lacks a comparison to these tools. For instance, Galiez et al. has a better accuracy than PhANNs for capsid and tail predictions (as indicated in Table 1). And while yes, PhANNs is considering more than just these two types of phage proteins, a comparison of how PhANNs performs relative to these other solutions would convince the reader that they should use PhANNs. Or perhaps they should use another tool for specifically capsid genes in addition to PhANNs. How does PhANNs predictions compare to these other tools? Is it classifying the same proteins?***

To compare PhANNs directly with the Galiez and Baldi’s VIRALpro, we ran our test set through the VIRALpro SVM and, conversely, ran VIRALpro’s test set through PhANNs. We discuss the results in lines (286-298). Both tools have similar performance, but in addition to classifying more functional classes, PhANNs is significantly faster as well as easier for the average non-computational biologist to use.

***-------------------------------------***

***Reviewer #2: The manuscript by Cantu et al. presents a method for classifying proteins into 11 categories: 10 phage structural types and one catch-all ‘other’ group. The method applies neural network training, and used a large number of protein sequences to generate 12 models. Their source code is freely available under the MIT license for anonymous download in Github, and there is a webserver that applies one of those models (tetra_sc_tri_p) to user data. This work extends upon previous work by the same group and is meant to fill a need in the field for quick classification methods of unstudied phage proteins with low likelihood to be anything besides phage structural proteins. There are multiple concerns, however, around the generation of the training set and validation of the resulting models.***

*GENERAL COMMENTS*

***1. The training data sets for the neural network models were collected from the NCBI database using the ncbi_get_structural.py script, which retrieves sequences from the NCBI protein database based solely on how they are named by the sequence depositors. This is problematic, as a) many phage protein annotations in the database are incorrect, and b) even if they are “correct” there is no enforceable naming convention between annotators. For example, the term “minor tail protein” could refer to baseplate components, tail needles, tail fibers, tail tape measure proteins or head-tail joining proteins. Likewise, some groups may use alternate names for the major capsid protein, such as “coat” or “MCP”, and some names may simply be misspelled. It should also be noted that the ncbi_get_structural.py script contains a typo at line 145 (“head-tail joinning”) that would have interfered with data retrieval****.*

We are aware of these limitations; nevertheless it has been shown in different machine learning applications that a larger number of training examples is more important for model performance than a perfectly curated training set^2^. PhANNs uses a much larger database than similar tools (2 or 3 orders of magnitude larger). We performed manual curation to address these limitations, and have now included a paragraph describing these points in the Methods section, under the heading named “The databases of “classes.””

Regarding the typo in the script: if a term is not found in the database, NCBI ignores it. Therefore, searching for “head-tail joinning” is equivalent to searching for “head-tail”. This was unintentional and this behavior will change if a misspelled protein is added to the database. We modified the script to match the intended search terms. We thank the referee for their attention to detail.

***2. Manual curation is mentioned in the manuscript (lines 25, 83, 93, Table 2), but no curation criteria for class inclusion or exclusion are provided. Did the curation involve any validation that the proteins in a given class (e.g., tail fiber) actually had their named functions? Some categories, like baseplate, decreased significantly after manual curation (Table 2) but we do not know why.***

Curation consisted of grouping sequences by their description (part of the fasta header) and deciding which descriptions to include. Details can be found here (<https://github.com/Adrian-Cantu/PhANNs/tree/master/model_training>) and the list of descriptors is included here (<https://github.com/Adrian-Cantu/PhANNs/tree/master/model_training/01_fasta>)

In the particular case of tail fiber, we did not include the descriptions “phage tail fiber assembly protein” (3,662 proteins), nor many “partial protein” variations (1,500+ proteins). As for baseplate we didn’t include any variation of “wedge” (4,000+ proteins) or “partial” (1,500+ proteins).

***3. The biological or structural definitions of each of the ten classes are not defined in the manuscript, so it is difficult to tell what structural components belong to each class. While terms such as “major capsid” and “portal” are generally well-agreed upon (and will often contain those terms in their name), other categories are more ambiguous. Aside from the ambiguity in what constitutes a “minor tail protein” described above, it is not clear what qualifies as “major tail” (could be tail tube, tail sheath or both), a “tail shaft”, or what the structural distinction is between “collar” and “head-tail joining” proteins. The authors are free to define the classes as they wish, but detailed definitions are required, especially if the intent is for these annotations to be applied to novel phage genomes.***

Descriptions/definitions of the ten structural classes are now provided at the beginning of the Methods section (lines 104 -117).

***4. The manuscript describes validation of the neural network against sets of database proteins, but there is no validation of the tool against sets of known, experimentally verified proteins, or against clusters of structural proteins that are supported by external bioinformatic evidence (e.g., conserved domains, COGs, high-quality UniProt/SwissProt annotations). The authors describe this as a tool for the annotation of novel phage genomes, so there must be some evidence that the tool “works” on predicting known proteins. This validation should also include a description of the scoring criteria produced in the tool output, which appears to score each protein for each category on a scale of 0 to 10. It is not clear how to interpret these outputs or what constitutes a “good” score for the tool.***

We added a new section to the manuscript (lines 189-203) that describes the interpretation of the PhANNs score and how to derive a classification and confidence from it. We also added two figures: Figure 6 shows the relationship between the PhANNs score and the confidence for each class, and Figure 7 shows the effect on accuracy of excluding low scoring predictions.

For each input sequence, PhANNs run 10 ANNs predictions (those trained during the 10-fold cross-validation). Each of those 10 ANNs outputs the soft-max scores for every class (a number between 0 and 1, such that the score of each class adds to 1). PhANNs outputs the per class sum of the ten ANNs scores (the PhANNs score). The input sequence is classified as the class with the highest PhANNs score, and is highlighted in yellow.

To give a clearer indication of the quality of this prediction we added a “Confidence” score to each prediction. The “Confidence” score shows what fraction of test set sequences with this or higher PhANNs score were correctly classified (true positives). For example, a sequence classified as “major capsid” with a PhANNs score of 7 has 97% confidence that the classification is correct, while a “Tail fiber” with a PhANNs score of 7 has only 82.4% confidence that the classification is correct.

To provide evidence that the tool works on novel phage proteins, we added additional validation testing using the VIRALpro test set, which is based on the Seguritan et al. article,^1^ which is itself based on the ACLAME database, a set of well-curated mobile genetic elements (including phages) and on RefSeq. Though PhANNs was not trained with this data (and hence the data is novel from PhANNs perspective), it had a similar performance with this test set as with its own test set. The results are summarized in **Table 7.**

*SPECIFIC COMMENTS*

*Line 43 – ‘by lysing specific components of microbiomes’ is unclear. Is it meant that specific taxa are targeted? Phage can also interact with bacteria in ways other than lysing them.*

We have modified the first paragraph of the Introduction for greater clarity. Indeed, lysis is only one of several possible mechanisms that affect phage-bacterial interactions.

*Lines 60-62 – the argument that phage therapy has increased demand for phage annotation is supported, but the implication here seems to be that annotation of structural proteins will provide higher confidence in using phages for therapy. Is there a specific connection (positive or negative) to structural proteins and phages used for therapy? What does “provisional” mean in this context? Is this just another word for “inaccurate”?*

In cases where a lytic phage is identified as potentially useful because it targets a relevant patient strain, knowing as much about the phage is very useful when distinguishing between toxins or virulence factors that may worsen/adversely affect the patient’s health and the relatively innocuous, non-toxic phage structural components. The most thorough descriptions of phages help when obtaining FDA permission for use of a phage as a therapeutic. Moreover, in the future, engineering of phages may become more common to target specific virulent bacteria that have developed resistance to available phages. In such cases, being able to predict the best components to mutate or evolve will be highly useful.

*Figs. 2, 3 and 4 are missing y-axis labels. The bars in Figs. 2 and 3 are very narrow and can only be viewed when zoomed in to >200%.*

We added Y-axis labels to Figures 2,3 and 4. Furthermore Figures 2 and 3 were split into two figures each to make them more readable. Two of the new figures are in Supplementary Materials.

Bibliography

1.	Seguritan, V. *et al.* Artificial Neural Networks Trained to Detect Viral and Phage Structural Proteins. *PLoS Comput. Biol.* **8**, (2012).

2.	Halevy, A., Norvig, P. & Pereira, F. The Unreasonable Effectiveness of Data. *IEEE Intell. Syst.* **24**, 8–12 (2009).
